# Restriction in functioning and quality of life is common in people 2 months after compensable motor vehicle crashes: prospective cohort study

**DOI:** 10.1186/s40621-015-0042-7

**Published:** 2015-05-21

**Authors:** Jagnoor Jagnoor, Annelies De Wolf, Michael Nicholas, Chris G Maher, Petrina Casey, Fiona Blyth, Ian A Harris, Ian D Cameron

**Affiliations:** 1 John Walsh Centre for Rehabilitation Research, University of Sydney, Sydney, Australia; 2 University of Sydney, Sydney, Australia; 3 The George Institute for Global Health, University of Sydney, Sydney, Australia; 4 University of New South Wales, Sydney, Australia

**Keywords:** Motor crash, Injury, Sub-acute outcomes, Compensable settings

## Abstract

**Background:**

We sought to identify the role of pre-injury socio-demographic and health characteristics, and injury severity in determining health-related quality-of-life outcomes for mild to moderate injuries 2 months after a motor vehicle crash in a compensable setting.

**Methods:**

People aged 17 years and older, injured with a New Injury Severity Score of 8 or less, in a motor vehicle crash in New South Wales and who had registered a claim with the Compulsory Third Party Insurance scheme from March to December 2010 were contacted to participate in the study. Information for 364 eligible participants was primarily collected through telephone interview, approximately 2 months after injury.

**Results:**

Substantial proportions of participants continued to have adverse outcomes approximately 2 months after their injury with mean Short Form Health Survey (SF-12) physical component score of 36.7 (SD ±10.3), SF-12 mental component score of 46.6 (SD ±11), Euro Qol (EQ) analogue scale score of 65.8 (SD ±18) and Euro Qol five dimension (EQ-5D) summary score of 0.70 (SD ±10). Key factors predicting adverse outcomes were prior chronic illness, obesity, hospitalisation and self-perceived threat to life due to injury.

**Conclusions:**

This study highlights the substantial impact of apparently “minor” motor vehicle crash injuries in a compensable setting and suggests targets for studies of tertiary prevention to improve health-related quality-of-life outcomes.

**Electronic supplementary material:**

The online version of this article (doi:10.1186/s40621-015-0042-7) contains supplementary material, which is available to authorized users.

## Background

There is limited information on health and quality-of-life outcomes, and factors influencing functioning and quality of life after a motor vehicle crash in a compensable environment, particularly for people with mild to moderate musculoskeletal injuries. Previous research on compensable mild to moderate musculoskeletal injuries has reported persisting poorer outcomes as compared to the general population (Gabbe et al. 2007; Zatzick et al. 2008; Carroll et al. 2008). Similar findings have also been reported in the context of work-related injuries (Haldeman et al. 2008; Lyons 2010).

About half of all injuries reported following motor vehicle crashes are whiplash-associated disorders. Older age, female gender, higher initial pain, greater number of symptoms, greater initial disability, passive coping style, depressive mood, fear of movement, helplessness, low expectation of recovery, impact on work status, and compensation are some of the factors predicting poorer health outcomes following whiplash-associated disorders (Carroll et al. 2008; Holbrook et al. 2001; Casey et al. 2011). A recent study on predictors of poor outcomes in a population of injured people from a range of causes including road transport injuries identified being female, prior chronic illness, injuries to multiple body regions, being hospitalised for injury, self-perceived threat to life and difficulty accessing health services as the key factors (Holbrook and Hoyt 2004).

In New South Wales (NSW), compensation from the Third Party Insurance scheme (also called Compulsory Third Party Insurance or CTP) is available for persons killed or injured as a result of a motor vehicle crash. The scheme does not cover the at-fault driver, although there are exceptions for people with very severe injuries for whom the Lifetime Care and Support scheme is available. The CTP scheme provides benefits for injured persons that include medical treatment, rehabilitation expenses, compensation for lost earnings and other crash-related expenses. The scheme is designed to support early treatment and recovery. In the 2011–2012 financial year, nearly 14,000 claims were made and payments to injured people were approximately AUD 1290 million (USD 1005 million) (Holbrook and Hoyt 2004). There is a huge socio-economic and health-related burden due to motor transport crashes. If we are to improve CTP scheme outcomes, a better understanding of the factors related to poorer health following mild to moderate injuries in a compensable environment is required to design and implement more effective interventions.

The NSW Lifetime Care and Support scheme is available for people with spinal cord injury, extremely severe traumatic brain injury or amputations sustained in a motor vehicle crash. Hence, the key interest of this research work is on less severe injuries (i.e. mild to moderate injuries). The primary aim of this paper is to describe the socio-demographic, health and injury characteristics; level of mental and physical functioning; and health-related quality of life (HRQoL) at 2 months after injury from a compensable motor vehicle crash. The secondary aim is to explore the role of pre-injury socio-demographic, health, lifestyle habits and injury-related characteristics on the participants’ health status 2 months after a motor vehicle crash.

## Methods

Participants were enrolled on average 2 months after injury and were followed up at 12 and 24 months. The 2 months time period is the earliest time that it was feasible to identify and recruit eligible participants from an insurance database. Results presented here report health outcomes 2 months after injury and explore the association with socio-demographic, health and injury characteristics. The outcome measures at 12 and 24 months will be reported separately.

### Study settings and study population

Potential participants were identified from the NSW Motor Accident Authority (MAA) Personal Injury Registry database. The MAA is the government regulator of companies providing third party motor vehicle crash insurance in NSW. This database contains data on people who made a compensation claim through one of two claim notification processes: the Accident Notification Form or a Personal Injury Claim Form. The Accident Notification Form is for a limited insurance claim that provides for the early payment of reasonable and necessary medical expenses, and/or lost earnings up to a maximum of AUD 5000, regardless of fault. It is completed and sent to the insurer within 28 days of the crash. The Personal Injury Claim Form is for a full insurance claim.

People aged 17 years and over, who had sustained injuries in a motor vehicle crash in NSW between March and December 2010, were identified through the database and invited to participate in the study. Participants were excluded if they sustained severe injuries (severe traumatic brain injury or spinal cord injury, or injury requiring hospitalisation for more than 7 days, or who had a New Injury Severity Score greater than 8) (Osler et al. 1997) or were unable to complete questionnaires by telephone in English, or if contact could not be initiated until 60 days or less after the crash date.

**Fig. 1:**
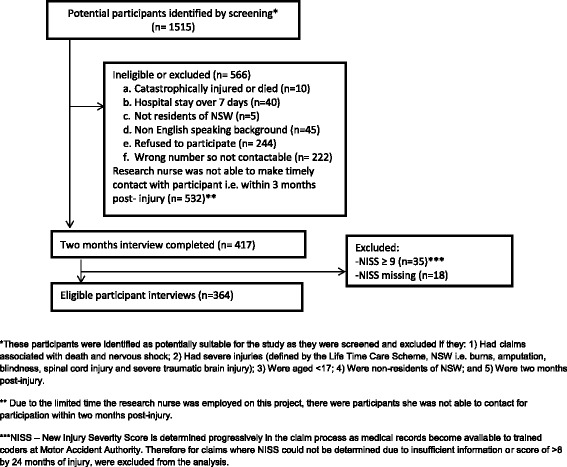
Flowchart for participation in the study

A total of 1515 eligible insurance claims were lodged between March 2010 and December 2010 (see Fig. 1). A letter of invitation was sent by the MAA together with the study Participant Information Sheet. An opportunity to opt out of the study was provided at this stage by calling at a designated study number. Potential participants were then contacted by telephone approximately 2 weeks later, and verbal consent was sought. The data was identifiable until follow-up interviews were completed. On completion of all follow-up interviews, data was de-identified. Stringent measures were taken to ensure confidentiality of data. Data files were saved on a secure server with access to only the research nurse and the study investigator. Data was de-identified for analysis. Of the 1515, 1098 were not eligible or refused to participate, and of the remaining 417 who participated in the 2 months interview, 53 were excluded as they had missing New Injury Severity Score (NISS) or an NISS >8 (those with severe injury). This left 364 participants that could be included in the analyses. The characteristics of study participants relative to those who were not contacted or refusing participation (Additional file 1: Table S1) were similar in age, sex distribution, injury severity and type of insurance claim made. The interview schedule was structured and used a closed question format to collect information on participant’s demographics, return to work, motor vehicle crash details and pain, disability and HRQoL. The interview took approximately 45 min, and all interviews were administered by one trained and experienced research nurse. To establish eligibility and demographics such as age, sex, injury severity score and country of birth, the Personal Injury Registry (PIR) database was used. All other variables were collected through telephone interview.

### Pre-injury socio-demographic and health characteristics

Socio-demographic details that were recorded were age, sex and highest level of education (primary, secondary, certificate, advanced diploma, bachelor, graduate and post-graduate) and reclassified into secondary education or less, post-secondary diploma or certificate, bachelor degree or more. Information was also collected on pre-crash work status and the person’s role in the crash.

Participants were asked to describe their general health status prior to the motor vehicle accident, using a five-point Likert scale (excellent, very good, good, fair or poor). Body mass index (BMI) was calculated from self-reported height and weight. BMI was classified according to WHO guidelines as BMI <20 (underweight), BMI 20–24.9 (normal), BMI 25–29.9 (overweight) and BMI ≥30 (obese) (World Health Organization 1998).

Smoking status was classified as current smoker daily/less than daily, ex-smoker daily/less than daily, and never smoked. For analyses, participants were identified as smokers if they were current smokers, daily or less than daily.

Chronic illness was determined by asking participants if they had been diagnosed in the last 3 months with any of the following: asthma, cancer, heart or circulatory condition, diabetes, arthritis, osteoporosis, mental and behavioural problems, neck and back problems/disorder, or pain. If participants identified with any illness of more than 3 months, then this was considered as having a chronic illness. Depression was identified if the participant reported clinically diagnosed depression or was on anti-depressant treatment. Participants were also queried about anxiety, if clinically diagnosed.

### Injury variables

Role of the injured person in the crash was classified as driver, passenger, rider, pillion, bicyclist or pedestrian. The Abbreviated Injury Scale (AIS) coding system was used to classify the participants into the mild (NISS 1–3) and moderate (NISS 4–8) groups based on the NISS (MacEachen et al. 2010). Severe and critical injuries (NISS score of >8) were excluded from the study. This injury coding is included in the MAA PIR database. Trained and experienced coders are used to code the reported injuries.

Admission to hospital for one or more nights following the injury was collected. Participants were asked to rate their self-perceived threat to life in the motor vehicle accident on a five-point scale (none, small, moderate, great, overwhelming).

### Outcome measures

#### Health status outcome

Health status outcomes were measured using the Medical Outcomes Survey Short Form-12 (SF-12 version 1 and Euro Qol five dimension three level, EQ-5D-3L) (Szende et al. 2007). The SF-12 measures general health (physical and mental health). Scoring of the SF-12 provides two component scores, the physical and mental component summary scores (PCS and MCS, respectively) which are standardised to a mean of 50 with a standard deviation of 10 (Elbers et al. 2013; Lippel 2007). We also used question 1 of the SF-12 “General health status” (“In general, how would you say your health is?” The answer options were excellent, very good, good, fair and poor) as an outcome measure.

The EQ-5D is a generic measure of health status and was selected to assess the functional outcome. The EQ-5D defines health along five dimensions that are mobility, self-care, usual activities, pain/discomfort, and anxiety/depression. Each dimension captures three levels of functioning, indicating no problems, some problems and extreme problems in the specified dimension. Disability and functioning is assessed based on EQ dimensions of mobility, self-care and usual activities. The single index value (EQ-VAS) records the respondent’s self-rated health on a vertical analogue scale where the endpoints are labelled “best imaginable health state” and “worst imaginable health state” (Murgatroyd et al. 2011).

Psychological injury such as phobic, anxiety disorders and depression episodes were derived based on the International Classification of Diseases and Injuries—Australian Modification (ICD-10-AM) coding and included codes F43, F06, F31, F32, F33, F40, F41, F43 and F91–F93 (see Additional file 2: Table S2 for details). These codes were derived from the PIR database.

### Other outcome measures

Return-to-work outcomes included “If you were employed before the accident, have you returned to work?” with answer options “yes full duties or yes modified duties (like reduced hours, lifting restrictions)” and days of work lost. Usual activities question included “Regardless of whether you were employed before the accident, have you returned to your usual activities?” with answer options “yes” or “no”.

### Statistical analyses

Descriptive statistics were used to summarise characteristics of the study participants. Differences in health status (PCS, MCS, EQ-5D summary score and EQ-VAS) were assessed using ANOVA test between pre-injury socio-demographic, pre-injury health characteristics, and injury variables. Linear and logistic regression models were employed to identify predictors of HRQoL measures or problems in EQ-5D dimensions measured, 2 months after injury. EQ-5D three levels were recoded into two categories (“no problem” or “some or extreme problem”). In the multivariate regression analyses, backward elimination approach was used to determine independent risk factors for HRQoL including self-reported general health. We progressively eliminated variables with the highest *P* value one at a time, retaining only those with *P* ≤ 0.05. Potential covariates assessed were age, gender, education level, marital status, paid work, self-rated health status prior to injury, chronic illness, chronic pain condition, depression prior to injury, role in crash, self-perceived threat to life, NISS, whiplash injury, fracture, BMI, smoking and hospital admission. SPSS version 21 was used for all statistical analyses (George and Mallery 2013).

Logistic regression analysis was used to determine the associations between pre-injury demographic (example employment status, income), health and socioeconomic factors; injury severity; and psychological reactions to the injury and its circumstances (catastrophising and self-perceived threat to life) and HRQoL at 2 months.

We also explored bootstrap stepwise regression for continuous outcomes to determine consistent predictors in accordance to the model presented here. We generated 1000 bootstrap samples and examined the proportion of samples in which the predictive factors were selected in final models after backward elimination. Almost all reported findings were present in more than 50 % of the samples, the only exceptions being chronic illness in the model for the EQ-5D VAS (present in 48.5 % of samples) and fracture in the models for EQ-5D usual activities and EQ-5D pain or discomfort (present in 41–47 % of samples). The bootstrapping suggested some additional findings in that BMI was selected in more than 70 % of samples for all models in Table 4 and in 69 % of samples in models for EQ-5D mobility; fracture was selected in 53 % of samples in models for SF-12 PCS; role was selected in 53–59 % of samples in models for SF-12 MCS, EQ-5D self-care and EQ-5D usual activities; health was selected in 60 % of samples in models for EQ-5D pain or discomfort and EQ-5D anxiety or depression; and age was selected in 67 % of samples for models of EQ-5D mobility.

### Ethics

The study was approved by the Health Research Ethics Committee of Concord Hospital (Sydney, Australia).

## Results

**Fig. 2:**
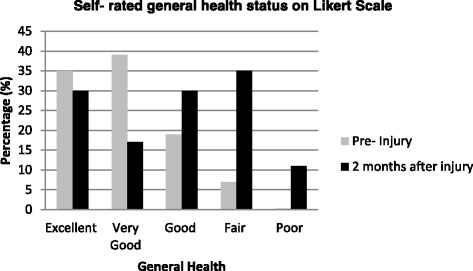
Self-rated health pre-injury and 2 months after injury

The characteristics of the study participants are reported in Table 1. About 63 % of the study participants were females with a mean age of 46 years, and 63 % were in paid employment and had a mean NISS of 2. Only 1 participant reported poor health pre-crash, and this increased to 44 (12 %) participants 2 months after injury (<0.0001) (Fig. 2).

**Table 1:** Socio-demographic characteristics of the phase 1 study participants (n = 364)

Characteristics	Participants *n* = 364 (%)
Age (years)	45.3 ± 16.7^a^
Sex (female)	229 (62.9)
Country of birth	
Australia	236 (64.8)
United Kingdom	24 (6.6)
Other	104 (28.6)
Marital status^b^	
Married/de facto	213 (58.5)
Divorced/widowed/separated	64 (17.6)
Single	86 (23.6)
Education level^b^ number (%)	
Bachelor of higher degree	100 (27.5)
Advanced diploma/certificate	95 (26.1)
Secondary education	160 (44.0)
Primary education	8 (2.2)
Pre-injury employment status prior to injury	
Paid employment/self employed	227 (62.3)
Non-paid voluntary work	2 (0.5)
Student	23 (6.3)
Home duties	25 (6.9)
Unemployed (health reasons)	20 (5.5)
Unemployed (other)	12 (3.2)
Retired	55 (15.1)
Hours worked prior to injury (hours/week; for those employed)	33.7 ± 13.0

^a^Mean ± standard deviation

^b^One missing data

Participants reporting affirmative self-perceived threat to life at the time of crash and history of chronic illness and/or depression were reported to have significantly poor health outcome scores (Table 2). A NISS score that rates the injury as more than mild (greater than 3), the presence of a fracture, one or more nights admitted to hospital and self-perceived threat to life are associated with one or more of the health outcomes (Table 3).

**Table 2:** SF 12 physical component score (PCS), mental component score (MCS) and EQ-5D-3L measures (summary score and EQ-VAS) stratified by pre-injury socio-demographic characteristics and health characteristics

		PCS		MCS		EQ-5D summary		EQ-VAS	
Pre-injury socio-demographic characteristics	*N*	Mean (SD)	*P* ^a^	Mean (SD)	*P* ^a^	Mean (SD)	*P* ^a^	Mean (SD)	*P* ^a^
Age (years)			0.003		0.37		0.008		0.17
<45	192	38.4 (11.7)		47.1 (10.8)		0.74 (0.16)		67.3 (21.4)	
≥45	171	34.8 (11.0)		46.0 (11.8)		0.69 (0.21)		64.1 (22.9)	
Gender			0.89		0.25		0.74		0.94
Male	135	36.8 (12.1)		47.5 (11.1)		0.72 (0.19)		65.9 (22.9)	
Female	229	36.6 (11.2)		46.1 (11.3)		0.71 (0.18)		65.7 (21.7)	
Marital status^b^			0.04		0.98		0.31		0.01
Married/de facto	213	36.7 (11.7)		46.5 (11.3)		0.71 (0.20)		66.1 (22.3)	
Divorced/widowed/separated	64	34.0 (11.6)		46.9 (11.1)		0.70 (0.20)		59.2 (26.1)	
Single	86	38.8 (10.5)		46.6 (11.5)		0.74 (0.13)		69.9 (17.2)	
Education^b^			0.01		0.10		0.02		<0.001
Post-secondary	195	38.1 (11.5)		47.5 (10.8)		0.74 (0.15)		69.4 (19.9)	
Secondary or less	168	35.1 (11.3)		45.6 (11.6)		0.69 (0.22)		61.8 (23.8)	
Prior paid work status			0.03		0.19		0.33		0.04
Yes	227	37.7 (11.8)		47.2 (10.7)		0.72 (0.18)		67.6 (20.5)	
No	137	35.0 (10.9)		45.6 (12.1)		0.70 (0.20)		62.7 (24.5)	
*Mn (mean); SD (standard deviation); PCS (physical component score); MCS (mental component score); F (one-way ANOVA F statistic); P (p-value); N (number)*									
Self-rated health prior to injury			0.002		<0.001		<0.001		<0.001
Excellent/very good	274	37.7 (11.5)		47.8 (10.5)		0.72 (0.16)		69.1 (20.3)	
Good	67	34.9 (11.0)		45.1 (12.7)		0.72 (0.21)		58.7 (25.4)	
Fair/poor	23	29.8 (9.8)		36.7 (11.1)		0.57 (0.25)		46.1 (18.2)	
Body mass index (kg/m^2^)			0.003		0.02		0.003		<0.001
<20	28	39.1 (11.4)		47.5 (9.8)		0.73 (0.11)		69.4 (16.1)	
20–24.9	128	38.8 (12.1)		49.0 (10.0)		0.76 (0.17)		71.2 (19.7)	
25–29.9	115	36.6 (11.1)		45.3 (12.6)		0.70 (0.19)		65.8 (20.6)	
≥30	93	33.3 (10.5)		44.6 (11.0)		0.67 (0.19)		57.2 (26.1)	
Smoking			0.57		0.02		0.36		0.15
Yes	52	35.8 (11.4)		43.3 (13.0)		0.69 (0.23)		66.3 (22.2)	
No	312	36.8 (11.5)		47.1 (10.9)		0.72 (0.18)		61.5 (21.1)	
Prior chronic illness			<0.001		0.06		<0.001		<0.001
Yes	146	33.1 (10.2)		45.3 (12.0)		0.67 (0.19)		59.8 (22.6)	
No	218	39.1 (11.7)		47.5 (10.6)		0.75 (0.17)		69.8 (21.0)	
Depression			0.25		<0.001		<0.001		<0.001
Yes	15	33.3 (11.7)		28.4 (9.8)		0.18 (0.15)		39.9 (21.3)	
No	349	36.8 (11.5)		47.4 (10.6)		0.74 (0.15)		66.9 (21.5)	

^a^ANOVA test

^b^One missing data

**Table 3:** SF 12 physical component score (PCS), mental component score (MCS) and EQ-5D-3L measures (summary score and EQ-VAS) stratified by injury characteristics

		PCS		MCS		EQ-5D summary score		EQ-VAS	
Pre-injury socio-demographic characteristics	*N*	Mean (SD)	*P* ^a^	Mean (SD)	*P* ^a^	Mean (SD)	*P* ^a^	Mean (SD)	*P* ^a^
NISS			0.002		0.40		0.004		0.04
1–3	310	37.5 (11.7)		46.8 (11.1)		0.73 (0.18)		66.7 (22.0)	
4–8	54	32.3 (9.5)		45.4 (12.3)		0.65 (0.17)		60.2 (22.1)	
Whiplash^b^			0.58		0.06		0.52		0.84
Yes	224	37.0 (11.5)		45.7 (11.4)		0.71 (0.18)		65.6 (22.2)	
No	139	36.3 (11.5)		48.0 (10.9)		0.72 (0.20)		66.1 (22.2)	
Fracture^b^			0.009		0.78		0.005		0.39
Yes	30	31.5 (9.6)		47.1 (12.0)		0.63 (0.17)		62.5 (21.0)	
No	333	37.2 (11.6)		46.5 (11.2)		0.72 (0.18)		66.1 (22.3)	
Role in the accident			0.64		0.14		0.91		0.50
Driver	241	37.1 (11.5)		46.0 (11.3)		0.72 (0.18)		65.6 (23.2)	
Passenger	67	36.2 (11.2)		46.3 (12.1)		0.70 (0.20)		63.3 (21.4)	
Motor cyclist (rider/pillion)	21	33.1 (12.1)		49.6 (9.2)		0.71 (0.14)		69.5 (14.8)	
Bicyclist	19	37.3 (12.7)		52.2 (10.8)		0.74 (0.15)		72.8 (15.7)	
Pedestrian	16	36.5 (11.1)		46.4 (8.7)		0.71 (0.22)		65.6 (23.6)	
Self-perceived danger of dying			0.007		0.001		0.001		<0.001
Yes	128	34.5 (11.1)		43.9 (11.7)		0.67 (0.20)		58.8 (23.8)	
No	236	37.9 (11.6)		48.1 (10.7)		0.74 (0.17)		69.6 (20.3)	
Admitted to hospital at night			<0.001		0.43		<0.001		0.03
Yes	69	32.0 (9.9)		45.6 (12.1)		0.62 (0.24)		60.5 (22.8)	
No	295	37.8 (11.6)		46.8 (11.1)		0.74 (0.16)		67.0 (21.8)	

^a^ANOVA test

^b^One missing data

ANOVA was performed to assess associations between pre-injury socio-demographic and general health status, SF-12 (mental and physical component scores), EQ-5D summary scores and EQ-VAS scores (Table 4). No association was found between age and gender with short-term quality-of-life outcomes. Lower education level, (poor/fair/good) self-rated pre-injury health, affirmative self-perceived threat to life, BMI greater than 24, presence of chronic illness and hospital admission of at least 1 day were all independent predictors for worse outcome on one or more HRQoL score 2 months after injury.

**Table 4:** Independent risk factors for short-term (2 months) health and functional outcomes following a motor vehicle crash injury

Independent variable	General health status (Likert scale 1 to 5)	SF-12 PCS	SF-12 MCS	EQ-5D summary score	EQ-5D VAS
	*β* ^a^ (95 % CI)	*β* ^a^ (95 % CI)	*β* ^a^ (95 % CI)	*β* ^a^ (95 % CI)	*β* ^a^ (95 % CI)
Pre-injury health status					
Excellent/very good	0		0	0	0
Good	−0.55 (−0.83, −0.27)		−3.4 (−6.3, −0.5)	0.03 (−0.02, 0.08)	−6.5 (−12.3, −0.6)
Fair/poor	−1.46 (−1.89, −1.03)		−11.6 (−16.2, −7.0)	−0.10 (−0.18, −0.02)	−16.2 (−25.5, −6.9)
Education level (≤ secondary)					−5.1 (−9.3, −0.8)
Chronic illness		−5.9 (−8.2, −3.6)		−0.05 (−0.10, −0.01)	−4.8 (−9.6, 0.0)
Chronic pain				−0.08 (−0.14, −0.02)	
Self-perceived threat to life	−0.33 (−0.55, −0.11)	−3.2 (−5.5, −0.8)	−3.8 (−6.1, −1.5)	−0.04 (−0.08, −0.01)	−10.5 (−14.9, −6.1)
NISS (4–8)		−4.4 (−7.6, −1.3)			
Fracture				−0.10 (−0.17, −0.03)	
Whiplash	−0.23 (−0.45, −0.00)		−3.0 (−5.3, −0.7)	−0.07 (−0.11, −0.03)	
Body mass index (kg/m^2^)					
<20	0				0
20–24	−0.17 (−0.58, 0.24)				−0.8 (−9.1, 7.5)
25–29	−0.45 (−0.87, −0.04)				−3.4 (−11.8, 4.9)
≥30	−0.53 (−0.95, −0.10)				−8.9 (−17.6, −0.3)
Admitted to hospital (≥1 night)	−0.38 (−0.65, −0.10)	−4.0 (−6.9, −1.0)		−0.10 (−0.15, −0.06)	
Model *R* ^2^	0.22	0.13	0.11	0.19	0.19

Wald chi-square tests for logistic models and partial *F* tests for linear regression models. Backward elimination approach was used to determine independent risk factors. The underlying assumption for the above model is normal distribution of the score data. To check the impact of non-normality, the models were re-run after Blom’s normalising transformation of the dependent variables. The great majority of findings were confirmed, the notable differences being that the coefficients fair/poor health in models for EQ-5D outcomes became marginally non-significant (*P* values 0.060 and 0.082), the coefficient for chronic illness in the model for EQ-5D VAS became more clearly significant (*P* = 0.018), and the coefficient for BMI >30 in the model for EQ-5D VAS became non-significant (*P* = 0.12)

^a^Adjusted for other covariates in the column

People who rated their pre-crash health as “fair or poor” 2 months after the crash had significantly lower scores on all health outcome measures compared to those rating their health as “excellent” or “very good” 2 months after the crash (Table 4).

Table 5 shows the results of logistic regression analyses. Fracture was associated with sixfold higher odds of experiencing difficulty in self-care and usual activities. History of chronic illness and pain-related conditions were associated with around two- to threefold higher odds of experiencing difficulties in two out of the five EQ-5D dimensions. Self-perceived threat to life was associated with twofold higher odds of experiencing anxiety and depression. Limitations in mobility by type of road users were not statistically significant. Participants with moderate versus mild injury severity had significantly greater difficulties in the self-care and usual activities, and hospitalisation of one night or more was associated with threefold higher odds of experiencing difficulty with mobility.

**Table 5:** Independent risk factors for short-term (2 months) EQ-5D dimension outcomes following a motor vehicle crash injury

	Mobility	Self-care	Usual activities	Pain/discomfort	Anxiety/depression
Independent variable	Odds ratio^a^ (95 % CI)	Odds ratio^a^ (95 % CI)	Odds ratio^a^ (95 % CI)	Odds ratio^a^ (95 % CI)	Odds ratio^a^ (95 % CI)
Role in the crash					
Driver	1.00				
Passenger	0.46 (0.21, 0.99)				
Motor cyclist (rider/pillion)	1.92 (0.70, 5.24)				
Bicyclist	0.73 (0.22, 2.42)				
Pedestrian	2.98 (0.97, 9.15)				
Marital status^b^					
Married/de facto			0.64 (0.35, 1.18)		
Others			0.30 (0.13, 0.68)		
Single			1.00		
Working for pay				2.09 (1.19, 3.67)	
Chronic illness			2.21 (1.31, 3.74)	3.09 (1.66, 5.76)	
Pain-related condition	3.47 (1.82, 6.63)				
Self-perceived threat to life			1.95 (1.15, 3.31)		2.17 (1.37, 3.45)
NISS (4–8)		2.11 (1.07, 4.16)	3.67 (1.43, 9.43)		
Whiplash				2.42 (1.38, 4.22)	
Fracture		6.17 (2.60, 14.6)	6.64 (1.45, 30.5)	3.37 (1.06, 10.7)	
BMI (kg/m^2^)					
<20		1.00	1.00		1.00
20–24.9		0.61 (0.22, 1.74)	0.34 (0.12, 1.00)		0.90 (0.35, 2.29)
25–29.9		0.79 (0.28, 2.24)	0.41 (0.14, 1.23)		1.94 (0.77, 4.88)
≥30		1.74 (0.63, 4.81)	0.75 (0.24, 2.32)		2.30 (0.89, 5.29)
Admitted to hospital (≥1 night)	3.06 (1.71, 5.48)				
Concordance index/AUC	0.71	0.73	0.72	0.68	0.66

Wald chi-squared tests for logistic models and partial *F* tests for linear regression models

^a^Adjusted for other covariates in the column

Participants with whiplash-associated disorders compared to those without whiplash-associated disorders had significantly lower scores in the SF-12 for PCS and MCS, and self-reported health status measures. BMI categories were associated with the individual EQ-5D dimensions, and being overweight (25–30) or obese (≥30) was associated with significantly lower SF-12 PCS scores.

More than half (205; 56.3 %) of the study participants were working at the time of crash. A mean of 5 working days and a median of 9 working days were lost due to injuries to the time of interview. No loss of working days was reported by 22 % (91), and 17 % (69) had lost more than 15 working days due to crash injuries. At 2 months, 62 % (258) of participants reported that they had not fully returned to their usual activities.

## Discussion

To understand the burden of injury, information about injury-related disability outcomes is required (Lyons 2010; Polinder et al. 2010). In this representative sample of people with compensable personal injury insurance claims following motor vehicle crashes, mild to moderate injuries were observed to have major impacts on physical and mental well-being and health-related quality of life in the early post-injury phase. The study participants had access to payments for care and rehabilitation, and loss of earnings. It is to be noted that the physical health as measured by PCS and the HRQoL as measured by EQ-5D in the study participants were well below those of the Australian general population (Tucker et al. 2010).

This study underlines the substantial impact of apparently “minor” motor vehicle crash injuries at a population level and suggests targets for studies of tertiary prevention of long-term morbidity and disability. The multivariate analyses demonstrate that pain, disability (EQ dimensions of mobility, self-care and usual activities) and, to a lesser extent, injury severity are the main factors associated with physical functioning, but this is also influenced by health and other factors present prior to injury. The results from this study are broadly similar to those from the Prospective Outcomes of Injury Study (POIS) in New Zealand, a population cohort study of all injuries, as most participants in the cohort were experiencing worse health status and increased disability compared to before injury, despite less than one-third reporting admission to a hospital because of their injury (Derrett et al. 2011). The study has also drawn attention to the substantial health and societal impact of apparently minor injuries that have not required hospital admission (Wilson et al. 2013).

A number of pre-injury, injury-related and psychological factors were associated with health status following injury from motor vehicle accidents. Self-perceived threat to life in the crash was identified as an independent predictor of mental health and quality of life in the early phase after injury. Poor or fair self-rated health prior to injury, presence of chronic illness and/or depression, and obesity were associated with two out of three health outcomes studied. Greater injury severity (as measured by NISS) greater than 3 was associated with poorer physical health status, and hospital admission for the injury was associated with poorer quality of life.

Psychological factors such as presence of self-perceived threat to life in the crash and pre-injury chronic mental health problems suggest that psychological interventions could have a positive impact on self-rated quality of life following motor vehicle injuries. Because these factors were measured at the same time as health status in the current analyses, the directions of these associations cannot be established. One explanation is that many of the abnormalities of the psychological factors are a direct consequence of the injuries sustained.

Also, physiological factors, presence of chronic illnesses, whiplash-associated disorders and injury severity were predictive for several of the health outcomes of interest such as the SF-12 MCS and PCS. This is intuitive as prior studies have shown that presence of chronic illnesses and moderately severe injuries can lead to activity limitations and participation restrictions (Spearing et al. 2012). Previous studies have also reported self-perceived threat to life as a predictor factor for post-traumatic stress disorder (PTSD) and poor HRQoL outcomes in trauma patients (Holbrook et al. 2001).

The cohort of the study comes from a compensable injury claims database. It can be argued that the poor HRQoL outcomes are due to the compensable nature of the injury, leading to reporting bias for secondary gain. However, there are alternate explanatory theories to poor health outcomes observed in a compensable setting including secondary victimisation (Murgatroyd et al. 2011; Lippel 2007; MacEachen et al. 2010).

We achieved good response rate (from a compensable cohort using telephone interviews). The POIS in New Zealand achieved a response rate of 66 % using similar methods (Derrett et al. 2011). We comprehensively assessed predictors and measured health and social outcomes for mild to moderate injuries.

There are a number of limitations of the analyses that have been presented. The sampling was based on response to the call made by the research nurse. Whilst multiple attempts were made, a significant proportion of participants could not be contacted within 2 months of injury. As stated above, the current report examines pre-injury variables which have been collected following the injury. There is likely to be recall bias from retrospective recall of pre-injury health status (Spearing et al. 2012). However, it is likely to be more appropriate than applying population norms to evaluate the effect of acute injury on health-related quality of life from the general population (Wilson et al. 2012). The planned analysis of 12- and 24-month follow-up assessments will permit further exploration of predictors and causal relationships. Another limitation is that people with severe injuries have been excluded as have people unable to communicate in English, and the findings cannot be generalised to these groups. Also, we cannot disregard residual confounding from unmeasured or unaccounted factors that could have influenced observed associations with health status outcomes such as role in the crash and liability status. Hence, a more comprehensive research investigating all potential factors associated such as liability, claims process, health service seeking and utilisation is needed to truly understand the role of health and socioeconomic factors.

It is unlikely that the recruitment method would have introduced a systematic sampling bias. It is to be noted that the study represents people in the Compulsory Third Party scheme in NSW. As reported in Additional file 1: Table S1, the characteristics of study participants and those who refused or were not contacted are similar to all people making claims with, as expected, children and adolescents not being included and very few people with severe injuries included. The proportion of female participants is higher as compared to males being more at risk of injury in the cohort. This is perhaps representative of the distribution of claimants in the scheme that had nearly 20 % more female claimants as compared to males. This could be associated with schemes with “at fault” exclusion criteria. Hence, more females may be eligible to claim. It is reported that women have poor HRQoL outcomes than men and are at higher risk for psychological morbidity after trauma. Since women are over-represented in our cohort, this could have led to lower scores on HRQoL scales (Holbrook et al. 2001).

However, the study is unlikely to have recruited a fully representative sample of “at fault” injured motor vehicle users. The large number of motor vehicle insurance claims in the jurisdiction studied (about 14,000 per year in NSW) indicates a substantial public health issue that requires careful consideration to reduce the health, social and economic impact. The study results signify the need to collect information on HRQoL information on a routine basis. The complex interaction of factors contributing to this impact suggests that single interventions, with the exception of primary preventative measures that reduce the number of crashes, are unlikely to have very large impacts. However, tertiary prevention through coordinated treatment and rehabilitation services, and careful and evidence-based claims management principles could have important impacts (Suissa et al. 2006).

The results from this study representing a cohort from a compensable setting will assist in designing future interventions in the Compulsory Third Party scheme. Further examination of longitudinal data from this cohort will clarify the role of socio-demographic and health characteristics and compensation status on the characteristic main determinants of health outcomes in motor vehicle crashes.

## Conclusions

In conclusion, the results highlight the substantial impact of apparently “minor” motor vehicle crash injuries for people involved in compensation and suggest targeted studies of tertiary prevention to improve health-related quality-of-life outcomes.

## Additional files

Additional file 1: Table S1. Comparison of study participants to all claimants in the Personal Injury Registry database of New South Wales. Additional file 2: Table S2. Details of ICD-10-AM codes for physiological injuries.

## Abbreviations

AIS: Abbreviated Injury Scale
BMI: body mass index
CTP: Compulsory Third Party Insurance
HRQoL: health-related quality of life
ICD-10-AM: International Classification of Diseases and Injuries—Australian Modification
MAA: Motor Accident Authority
MCS: mental component score
NISS: New Injury Severity Score
NSW: New South Wales
PCS: physical component score
PIR: Personal Injury Registry
POIS: Prospective Outcomes of Injury Study
SF: Short Form Health Survey
WHO: World Health Organization
