# Supplementary material for: Restriction in functioning and quality of life is common in people 2 months after compensable motor vehicle crashes: prospective cohort study
Source: Inj Epidemiol. 2015 May 21;2(1):8. doi: 10.1186/s40621-015-0042-7 (PMC5005668; doi:10.1186/s40621-015-0042-7)
Supplement: Additional file 1: Table S1. — Comparison of study participants to all claimants in the Personal Injury Registry database of New South Wales. [file 40621_2015_42_MOESM1_ESM.doc]

Appendix 1 Comparison of study participants and all people sustaining injuries in motor vehicle crashes in NSW

The tables below compare the participants in the Phase1 Study database (N=364) with the claimants in the MAA database (N=1518) and the claimants who did not participate in the study (N=1101).

The tables show that the Phase 1 study group is similar in age, gender, injury severity, claim type, liability status, and insurer when compared with the claimants who did not participate (None Study) and the claimants presented in the MAA database as a whole.

Therefore this analysis has shown approximately a quarter (27%) of all people with CTP claims during the period of recruitment were recruited and those recruited were representative of all people with claims who met the inclusion criteria for the study.

| **Age and gender** | | |  |  |
| --- | --- | --- | --- | --- |
|  | MAA  Database | In Study | Not Included | P value |
|  | N=1518 | N=364 | N=1101 |  |
| Age (yrs) Mean (SD) | 43 ± 17 | 45.3 ± 16.7 | 42 ± 16 | <0.0001 |
| Gender N (%) |  |  |  | <0.001 |
| Female | 891 (59) | 229(63) | 635 (58) |  |
| Male | 623 (41) | 135 (37) | 460 (42) |  |

| **Claim type** N(%) | | |  | <0.0001 |
| --- | --- | --- | --- | --- |
|  | MAA | Phase1Study | None Study |  |
|  | N=1518 (%) | N=364 (%) | N=1101(%) |  |
| ANF claim | 672 (44) | 163 (45) | 484 (44) |  |
| ANF at fault | 91 (6) | 27 (8) | 59 (5) |  |
| ANF converted to full | 575 (38) | 130 (36) | 427 (39) |  |
| Direct full claim | 180 (12) | 44 (11) | 131 (12) |  |
| **Injury Severity** | | | | <0.0001 |
|  | MAA | Phase1Study= | None Study^1^ |  |
|  | N=1260 | N=364 | N=919 |  |
| ISS median (IQ) | 2 (2) | 2 (2) | 2 (2) |  |
| NISS median (IQ) | 2 (2) | 2 (2) | 2 (2) |  |
| ISS/NISS codes are missing N=180 | | | |  |
