# Supplementary material for: Restriction in functioning and quality of life is common in people 2 months after compensable motor vehicle crashes: prospective cohort study
Source: Inj Epidemiol. 2015 May 21;2(1):8. doi: 10.1186/s40621-015-0042-7 (PMC5005668; doi:10.1186/s40621-015-0042-7)
Supplement: Additional file 2: Table S2. — Details of ICD-10-AM codes for physiological injuries. [file 40621_2015_42_MOESM2_ESM.doc]

Appendix 2: Details of ICD 10-AM codes for physiological injuries.

| **ICD 10- AM code** | **Description** |
| --- | --- |
| F06 | Other mental disorders due to brain damage and dysfunction and to physical disease |
| F31-F33 | Depressive episodes; bipolar disorders, current episode depressed or mixed; recurrent depressive disorders |
| F40-F41 | Phobic anxiety and other anxiety disorders |
| F43 | Reactions to severe stress including acute stress reactions |
| F91-F93 | Conduct disorders, mixed disorders of conduct and emotions, emotional disorders with onset specific to childhood. |
